# Relationship between alveolar-bone morphology at the mandibular incisors and their inclination in adults with low-angle, skeletal class III malocclusion—A retrospective CBCT study

**DOI:** 10.1371/journal.pone.0264788

**Published:** 2022-03-01

**Authors:** Cai-Lian Lu, Bo-Wen Li, Mi Yang, Xiao-Qin Wang

**Affiliations:** 1 Department of Stomatology, The First Hospital of Shanxi Medical University, Taiyuan, China; 2 School and Hospital of Stomatology, Shanxi Medical University, Taiyuan, China; Universidade Federal Fluminense, BRAZIL

## Abstract

**Objective:**

To quantitatively study the effect of the labial inclination of the mandibular central incisors on the surrounding cortical and cancellous-bone morphology among patients with low-angle, skeletal class III malocclusion, by using cone-beam computed-tomography (CBCT) imaging.

**Materials and methods:**

The CBCT images of 60 patients with low-angle, skeletal class III malocclusion were divided into lingual-inclination, upright, and labial-inclination groups. The height of the alveolar bone and the thickness and area of the cortical, cancellous, and total alveolar bone were measured separately on each side of the mandibular central incisors.

**Results:**

The thickness of the labial cortical bone from 6 mm below the cementoenamel junction (CEJ) to the root apex; the thickness of the labial cancellous bone at the root apex; the total thickness of the alveolar bone at the root apex; the area of labial cortical bone; the total area of labial alveolar bone; and the height of the labial alveolar bone were highest in the labial-inclination group (all P<0.05). All these variables were positively correlated with the labial inclination of the mandibular central incisors (all P<0.05). There were no statistical differences between the groups for any of the measurements on the lingual side of the teeth (P>0.05).

**Conclusion:**

The morphology of the alveolar bone on the labial but not the lingual side of the mandibular central incisors was statistically significantly correlated with the labial inclination of those teeth in patients with low-angle, skeletal class III malocclusion.

## Introduction

Researchers overwhelmingly agree that the alveolar bone of the mandibular central incisors in patients with skeletal class III malocclusion is thinner than that in patients with skeletal classes I and II malocclusion [1, 2]. Compensatory or decompensatory treatment for skeletal class III malocclusion usually requires extensive movement of the mandibular central incisor. However, the range of orthodontic tooth movement and bone remodeling that can be achieved is restricted by the alveolar-bone morphology; which is known as the “anatomical boundary” [3]. Beyond this boundary, the alveolar bone may not undergo complete remodeling, resulting in contact of the root with the cortical bone. Such contact may lead to fenestration, dehiscence, root resorption, and an increased need for orthodontic anchorage [4]. Therefore, the thickness of the alveolar bone of the mandibular central incisors should be evaluated prior to orthodontic treatment.

Recent research in the field has mainly focused on the influence of different bone types on the thickness of the alveolar bone, or the influence of the change in incisal inclination before and after orthodontic treatment on the morphology of the alveolar bone. However, such studies are mostly limited to the apical area, and most measurements do not distinguish between cortical and cancellous bone. There have been few studies on the effect of labial inclination of the mandibular incisors on alveolar-bone thickness without orthodontic treatment, and there is a lack of data on the effect of cortical- and cancellous-bone area on alveolar-bone thickness. The morphology of the alveolar bone is traditionally evaluated with two-dimensional X-ray imaging, although such evaluation is limited around the anterior teeth [5, 6]. In recent years, the application of cone-beam computed tomography (CBCT) has been expanding in the field of dentistry and is regarded as an accurate tool for positioning and measurement [7]. It is a reliable source of data for clinical indicators that are difficult to evaluate, such as alveolar-bone height and thickness.

Previous studies [2] have confirmed that the alveolar bone of patients with high-angle, skeletal class III malocclusion is thinner than that in patients with low-angle malocclusion. We aimed to expand on those results by using CBCT to measure the thickness and height of the alveolar bone around the mandibular central incisors in patients with low-angle, skeletal class III malocclusion with different labial inclinations. The purpose was to determine whether the alveolar-bone thickness and height around the root of the mandibular central incisors differ based on labial inclination.

## Materials and methods

### Patients

This study was conducted in accordance with the Declaration of Helsinki, and the study was approved by the Medical Ethics Committee of the First Hospital of Shanxi Medical University (approval no. K085). All patients provided written informed consent for participation. All experiments were performed in accordance with relevant guidelines and regulations. The size of the population was predetermined by means of power analysis in IBM SPSS Statistics for Windows, version 22.0 (IBM Corp., Armonk, NY, USA). With a 1:1:1 group allocation ratio, a total sample size of 60 patients would yield more than 80% power (actual power, 0.944) to detect significant differences with a 0.5138 effect size at the α = 0.05 significance level among the three groups. The researchers retrospectively screened CBCT images that were archived in the First Hospital of Shanxi Medical University from June 2020 to November 2021. The study participants were 34 men and 26 women, age between 18 and 35 years, with an average age of 22 ± 3.93 years. The inclusion criteria were as follows: (1) skeletal class III malocclusion, -4°≤ ANB ≤ 1°; (2) MP-FH < 28°; (3) between 15 and 35 years of age; (4) no history of orthodontic treatment; (5) no periodontal disease; (6) no anterior-dentition defects or hyperdontia; (7) no history of anterior-tooth trauma; (8) complete root development, no obvious root resorption, no obvious curvature of the roots of the anterior teeth, and no history of incisal root-canal treatment; (9) crowding of the lower arch < 4 mm; and (10) no evident facial asymmetry or cleft lip and/or palate.

### Grouping

The patients were divided into three groups according to the mandibular central incisor-mandibular plane (L1-MP) angle: the lingual-inclination group, L1-MP < 85.6° (20 cases); the upright group, L1-MP 85.6°–99.6° (20 cases); and the labial-inclination group, L1-MP > 99.6° (20 cases). Patient information is summarized in Table 1, and there were no differences in any of the indicators except for labial inclination.

**Table 1:** Patient characteristics in different groups.

Characteristics	Lingual-inclination group (n = 20)	Upright group (n = 20)	Labial-inclination group (n = 20)	F	P
**Sex(F/M)**	12/8	8/12	6/14	3.801	0.150
**Age(years)**	20.40±4.16	21.95±4.12	22.75±3.37	1.876	0.163
**ANB (°**)	-2.81±1.21	-2.32±2.15	-1.69±0.72	2.868	0.065
**FMA (°**)	19.23±2.00	19.03±2.00	17.94±1.49	2.814	0.068
**L1-MP (°**)	79.47±2.59	89.78±2.33^a^	104.74±3.04^ab^	452.758	<0.001**

Values presented as mean ± standard deviation.

ANB, subspinale-nasion-supramentale angle; FMA, angle between the Frankfort horizontal plane and the mandibular plane; L1-MP, mandibular central incisor-mandibular plane angle.

** Statistically significant at P < 0.001.

### Measurement method

The original-volume CBCT images of the 60 patients were obtained by professionals from the First Hospital of Shanxi Medical University, by using a NewTom CBCT scanner (VG10048S; Verona, Italy). The CBCT parameters were as follows: voltage, 110 kV; scanning time, 3.6 s; scanning field, 15 cm × 15 cm; voxel size, 0.3 mm; and screen resolution, 1280px × 1024 px. The Digital Imaging and Communications in Medicine data of each patient’s CBCT image were imported into Dolphin Imaging software version 11.8 (Dolphin Imaging & Management Solutions, Oakdale, CA, USA) for three-dimensional reconstruction. The reconstructed slice thickness was set to 0.5 mm, and the patient’s head was calibrated according to the FH plane and the coronal plane, which was perpendicular to the FH plane and passed through the root points of the zygomatic arch on both sides.

**Fig 1:**
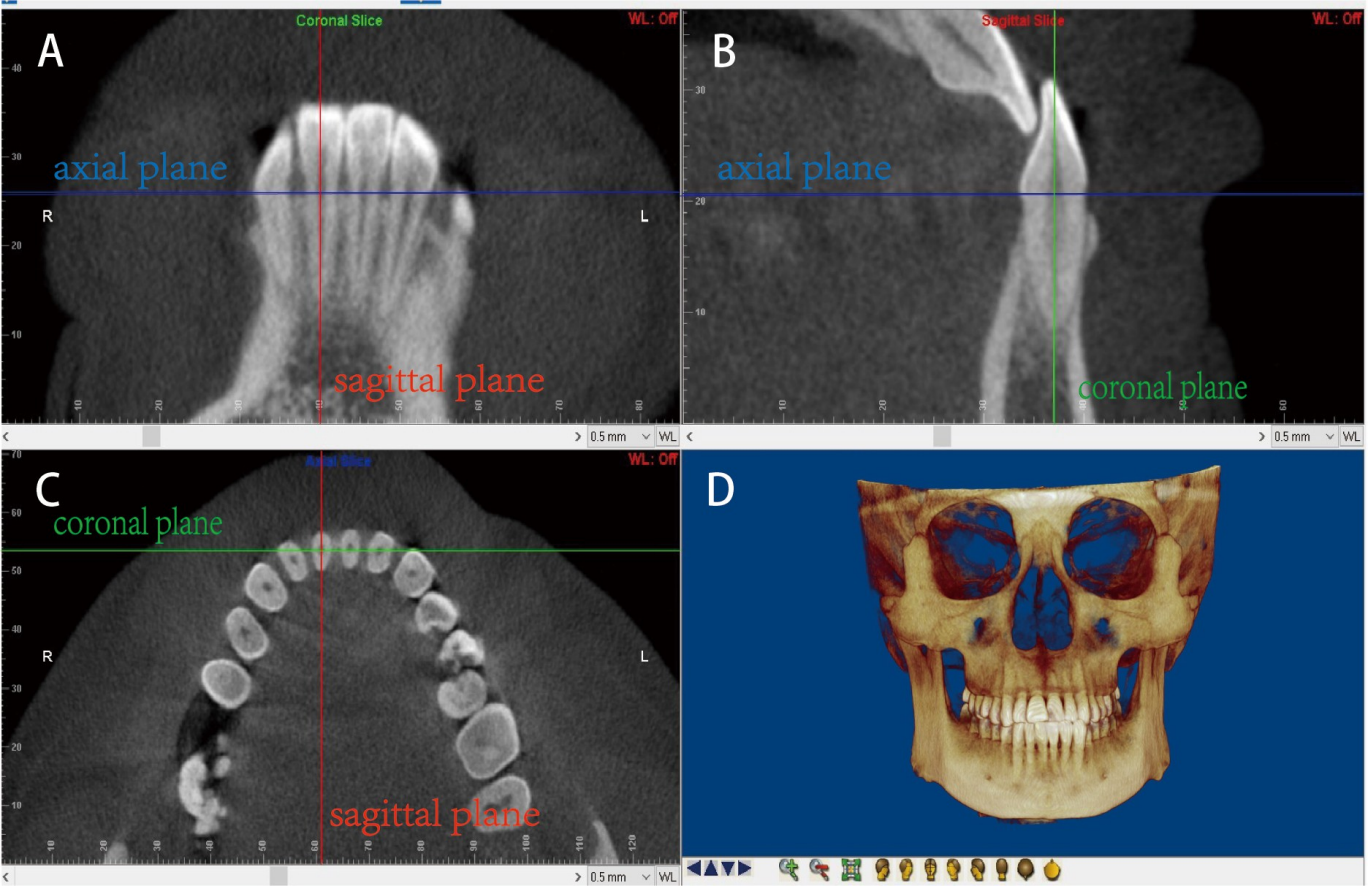
Locating the measurement planes for three-dimensional reconstruction. Locating the measurement planes for three-dimensional reconstruction. A: Coronal slice; B: sagittal slice; C: axial slice; D: reconstructed image. The planes are represented as three differently colored lines: red, sagittal plane; blue, axial plane; and green, coronal plane. Step 1: the blue line was adjusted on both the coronal and sagittal slices to overlap with the cementoenamel junction (CEJ) line, to obtain the axial slice. Step 2: in the axial slice, the intersection of the red and green lines was adjusted to the center of the pulp cavity of the cutting plane of the mandibular central incisor, and the image was rotated until the intersection of the red line and the measured tooth was the shortest. Step 3: the sagittal slice was rotated until the green line passed through the root apex and the midpoint of the CEJ. The coronal slice was rotated until the red line passed through the midpoints of the incisal edge and root apex. Finally, the sagittal slice of the desired tooth position was obtained.

**Fig 2:**
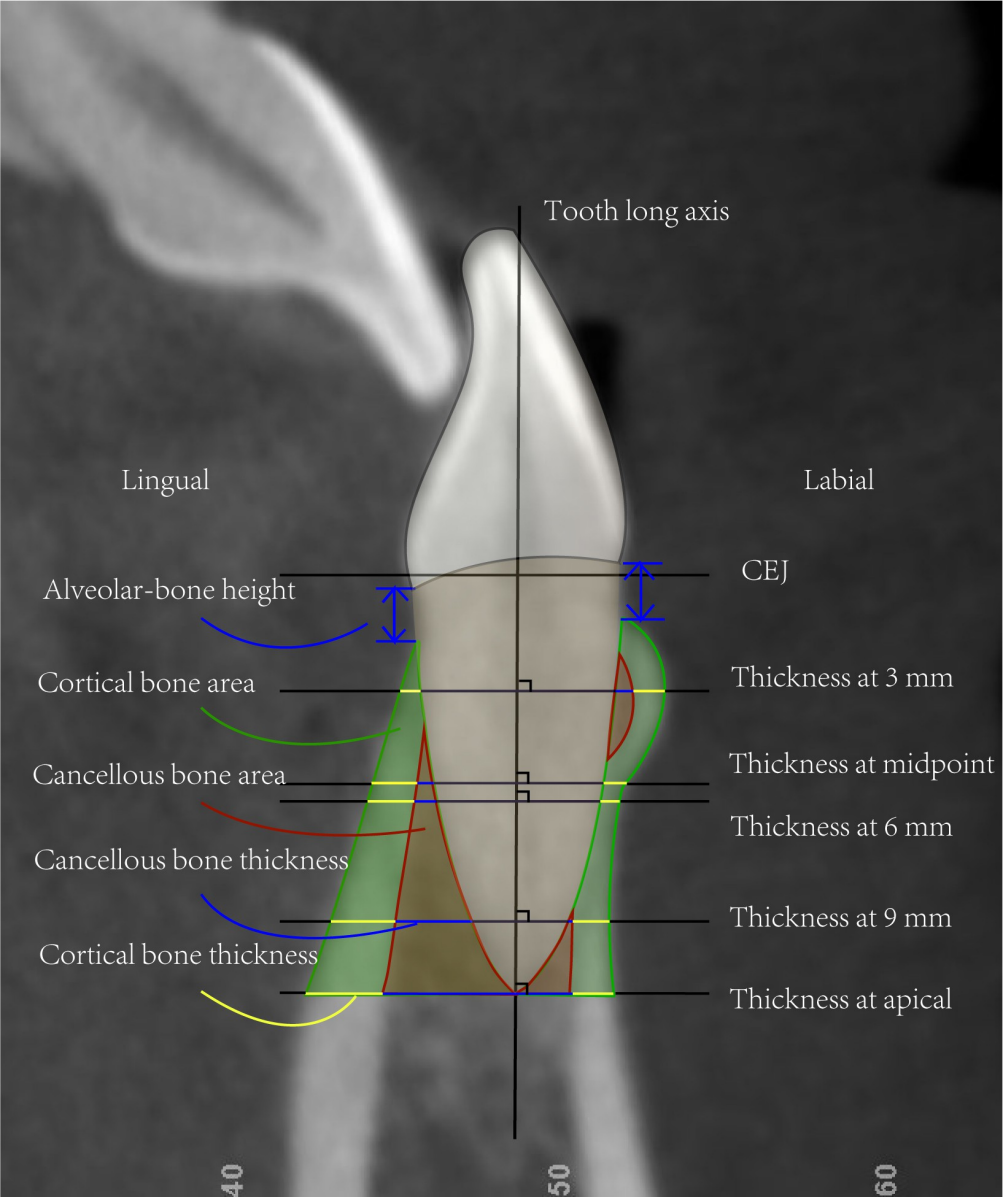
Measurement variables. The long axis of the tooth was defined as the line connecting the midpoint of the CEJ and the root apex. Five reference lines perpendicular to the dental long axis were established at the following positions: 3 mm, 6 mm, and 9 mm below the CEJ; the midpoint between the CEJ and root apex; and the root apex. The landmarks were obtained at the points where each reference line intersected with the medial and lateral sides of the cortical bone.

Fig 1 illustrates the method used to locate the measurement planes for three-dimensional reconstruction. The sagittal plane was usually selected as the observation plane, centered on the maximum labiolingual diameter of the target tooth. The height, thickness, and area of the alveolar bone of the mandibular central incisor were measured with the 2DLine and 2DSliceArea modules. The reference lines and landmark points were determined as explained in Fig 2. The survey items were defined as in Table 2.

**Table 2:** Definitions of each survey item.

Survey item	Define
**Alveolar-bone height**	The distance from the CEJ to the alveolar crest parallel to the long axis of the tooth.
**Cortical-bone thickness**	The distance between the intersections of the horizontal reference line with the inner and outer surfaces of the cortical bone.
**Cancellous-bone thickness**	The distance between the intersections of the horizontal reference line with the root surface and the inner surface of the cortical bone.
**Alveolar-bone thickness**	The sum of the cancellous- and cortical-bone thickness.
**Total thickness of labial and lingual alveolar bone**	The distance between the intersections of the horizontal reference line with the outer surfaces of the labial and lingual cortical bones.
**Cancellous-bone area**	The area enclosed by the apical plane, the root surface, and the outer surface of the cancellous bone.
**Cortical-bone area**	The area enclosed by the apical plane, the outer surface of the cancellous bone, and the outer surface of the cortical bone.
**Total alveolar-bone area**	The sum of the cancellous- and cortical-bone areas.

### Data collection and statistical methods

All patients were examined by two surveyors on the same computer. Paired t-tests were performed and intraclass correlation coefficients (ICCs) were calculated to assess systematic and random errors. There were no significant differences between the two measurements. The ICC between the two surveyors indicated high reliability (ICC, 0.95). The average of the two measurements was used for each patient.

All data were statistically analyzed by using IBM SPSS Statistics. Variables with a normal distribution were expressed as means ± standard deviations. The thickness, height, and area of the alveolar bone in the mandibular central incisor were compared among the three groups of patients by using one-way analysis of variance, and the least significant difference t-test was used for pairwise comparison between groups. The correlation between labial inclination and alveolar-bone morphology of the mandibular central incisor was evaluated by employing the Spearman correlation test. Results were deemed statistically significant when P < 0.05.

## Results

There were no significant differences in alveolar-bone thickness, height, or area between the left and right mandibular central incisors (P > 0.90). Therefore, the data for the left and right mandibular central incisors were combined.

### Comparison of cortical-bone thicknesses among the groups

The cortical-bone thicknesses atf the mandibular central incisors were compared among the three groups (Table 3). Except at 3 mm below the labial CEJ, there were significant differences in the thicknesses of the cortical bone on the labial side among the three groups at 6 mm below the CEJ, 9 mm below the CEJ, the midpoint between the CEJ and the root apex, and at the root apex (P < 0.05). Specifically, the cortical-bone thickness of the labial-inclination group was highest, followed by those of the upright and lingual-inclination groups. However, there were no significant differences in the thicknesses of the cortical bone on the lingual side among the three groups (P > 0.05).

**Table 3:** Comparison of cortical-bone thicknesses (mm) among the groups.

Side	Position	The lingual-inclination group	The upright group	The labial-inclination group	F	*P*
**Labial**	At 3 mm below CEJ	0.65±0.32	0.75±0.21	0.78±0.19	1.548	0.221
	At 6 mm below CEJ	0.47±0.20	0.69±0.31^a^	0.98±0.42^a^^b^	12.499	<0.001**
	At 9 mm below CEJ	0.88±0.27	1.25±0.51^a^	1.60±0.73^a^^b^	8.958	0.001*
	At the midpoint between the CEJ and the root apex	0.45±0.20	0.61±0.19^a^	0.88±0.29^a^^b^	17.250	<0.001**
	At the root apex	1.36±0.36	1.67±0.42^a^	2.02±0.42^a^^b^	13.596	<0.001**
**Lingual**	At 3 mm below CEJ	0.95±0.41	0.74±0.26	1.01±0.52	2.229	0.117
	At 6 mm below CEJ	1.84±0.63	1.47±0.49	1.68±0.60	2.094	0.133
	At 9 mm below CEJ	2.46±0.64	2.21±0.43	2.27±0.58	1.107	0.338
	At the midpoint between the CEJ and the root apex	1.76±0.72	1.52±0.44	1.70±0.70	0.752	0.476
	At the root apex	2.60±0.35	2.67±0.50	2.70±0.52	0.253	0.777

Values are presented as mean ± standard deviation.

CEJ, cementoenamel junction.

*Statistically significant at P < 0.01

**Statistically significant at P < 0.001.

^a^ Compared with the lingual-inclination group at the same site, P< 0.05

^b^ Compared with the upright group at the same site, P < 0.05.

### Comparison of cancellous-bone thickness among the groups

The thicknesses of the cancellous bone at the mandibular central incisors were compared among the three groups (Table 4). There were significant differences in the thicknesses of the cancellous bone on the labial side only at the root apex among the three groups (P < 0.05). Specifically, it was thickest in the labial-inclination group, followed by the upright and lingual-inclination groups. In addition, there were no significant differences in the thicknesses of the cancellous bone on the lingual side among the three groups (P > 0.05).

**Table 4:** Comparison of cancellous-bone thicknesses (mm) among the groups.

Side	Position	Lingual-inclination group	Upright group	Labial-inclination group	F	*P*
**Labial**	At 3 mm below CEJ	0	0.03±0.09	0	2.111	0.130
	At 6 mm below CEJ	0	0.07±0.19	0	2.813	0.068
	At 9 mm below CEJ	0.38±0.45	0.31±0.48	0.68±0.61	2.940	0.061
	At the midpoint between the CEJ and the root apex	0	0.07±0.20	0	2.097	0.132
	At the root apex	1.29±0.51	2.15±1.21^a^	2.85±129^a^^b^	10.735	<0.001**
**Lingual**	At 3 mm below CEJ	0	0	0	-	-
	At 6 mm below CEJ	0.16±0.22	0.25±0.30	0.18±0.31	0.556	0.576
	At 9 mm below CEJ	1.19±0.74	1.00±0.53	1.20±0.77	0.514	0.601
	At the midpoint between the CEJ and the root apex	0.09±0.22	0.25±0.32	0.11±0.25	2.152	0.126
	At the root apex	2.81±1.24	3.36±0.74	2.84±0.83	2.053	0.138

Values presented as mean ± standard deviation.

**Statistically significant at P < 0.001.

^a^ Compared with lingual-inclination group at the same site, P< 0.05

^b^ Compared with upright group at the same site, P< 0.05.

### Comparison of total alveolar-bone thicknesses among the different groups

The total alveolar-bone thicknesses at the mandibular central incisors were compared among the three groups (Table 5). There was a significant difference only at the root apex among the three groups (P < 0.05). Specifically, the alveolar bone at the root apex was thickest in the labial-inclination group, followed by those in the upright and lingual-inclination groups. There were no significant differences at other positions (P > 0.05).

**Table 5:** Comparison of total alveolar-bone thicknesses (mm) among the different groups.

	Lingual-inclination group	Upright group	Labial-inclination group	F	*P*
**At 3 mm below CEJ**	6.80±0.34	6.86±0.56	7.11±0.44	2.799	0.069
**At 6 mm below CEJ**	7.11±1.02	7.13±1.01	7.74±0.88	2.671	0.078
**At 9 mm below CEJ**	7.84±1.58	7.98±1.62	8.13±3.07	0.087	0.917
**At the midpoint between the CEJ and the root apex**	7.06±0.74	6.96±0.81	7.47±0.90	2.135	0.128
**At the root apex**	8.12±1.76	9.23±1.77^a^	10.50±1.74^a^^b^	9.217	0.001*

Values presented as mean ± standard deviation.

*Statistically significant at P < 0.01.

^a^ Compared with lingual-inclination group at the same site, P< 0.05

^b^ Compared with upright group at the same site, P< 0.05.

### Comparison of alveolar-bone areas among the different groups

The areas of the alveolar bone at the mandibular central incisors were compared among the three groups (Table 6). There were significant differences in the areas of the cortical bone on the labial side and the total alveolar bone among the three groups (P < 0.05). Specifically, the areas were largest in the labial inclination group, followed by those in the upright and lingual-inclination groups. There were no significant differences in the areas of the cancellous bone on the labial side nor the alveolar bone on the lingual side among the three groups (P > 0.05).

**Table 6:** Comparison of alveolar-bone areas (mm^2^) among the different groups.

Side		Lingual-inclination group	Upright group	Labial-inclination group	F	*P*
**Labial**	Total area of alveolar bone	8.22±1.84	13.56±5.14^a^	15.89±2.93^a^^b^	24.186	<0.001**
	The area of cortical bone	6.58±1.93	10.82±4.60^a^	13.17±2.01^a^^b^	23.140	<0.001**
	The area of cancellous bone	1.64±0.97	2.74±2.84	3.26±2.18	3.007	0.057
**Lingual**	Total area of alveolar bone	22.75±9.39	24.76±6.74	22.70±6.99	0.457	0.636
	The area of cortical bone	17.91±7.61	17.29±4.57	17.26±5.75	0.072	0.931
	The area of cortical bone	5.65±3.88	7.75±4.41	5.56±3.99	1.987	0.147

Values presented as mean ± standard deviation.

**Statistically significant at P < 0.001.

^a^ Compared with lingual-inclination group at the same site, P< 0.05

^b^ Compared with upright group at the same site, P< 0.05.

### Comparison of alveolar-bone heights among the different groups

Table 7 summarizes the differences in alveolar-bone heights among the different groups. A higher value indicates a greater loss of alveolar-bone attachment, that is, a lower alveolar-bone height. The alveolar-bone heights on the labial side of the mandibular central incisors differed significantly among the different groups (P < 0.05). Specifically, the bone was highest in the labial-inclination group, followed by those in the upright and lingual-inclination groups. There were no significant differences in alveolar-bone heights on the lingual side among the three groups (P > 0.05).

**Table 7:** Comparison of alveolar-bone heights (mm) among the different groups.

Side	Lingual-inclination group	Upright group	Labial-inclination group	F	*P*
**Labial**	1.33±0.37	1.81±0.53^a^	2.15±0.62^a^^b^	12.650	<0.001**
**Lingual**	1.58±0.50	1.55±0.30	1.54±0.48	0.045	0.956

Values presented as mean ± standard deviation.

**Statistically significant at P < 0.001.

^a^ Compared with lingual-inclination group at the same site, P< 0.05

^b^ Compared with upright group at the same site, P< 0.05.

### Correlation analysis of alveolar-bone morphology and inclination of the lower anterior teeth

**Fig 3:**
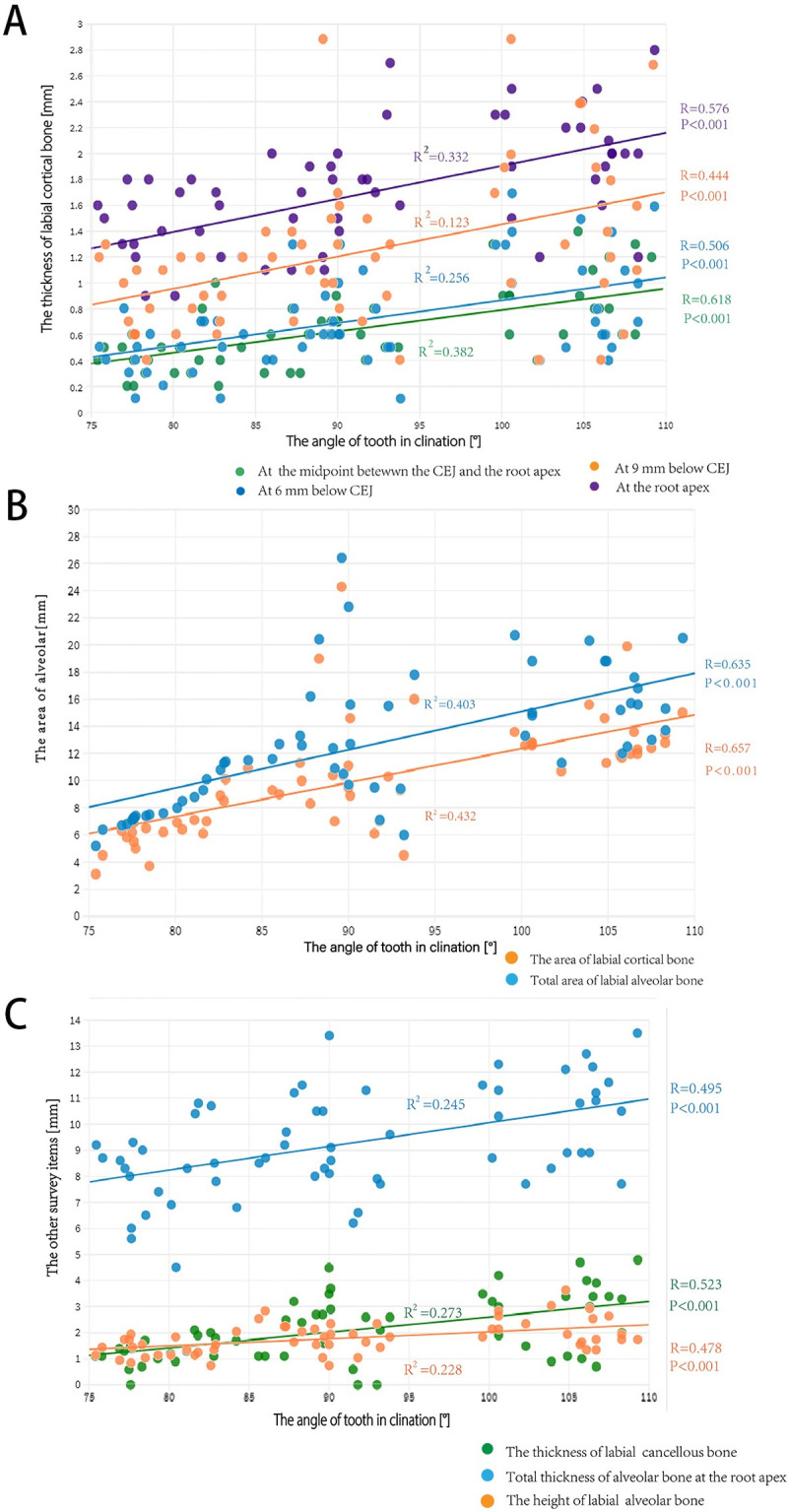
Scatter plots of survey items. A, labial cortical-bone thickness at 6 mm below the CEJ, 9 mm below the CEJ, the midpoint between the CEJ and the root apex, and at the root apex. B, cortical-bone area and total alveolar-bone area on the labial side. C, cancellous-bone thickness, total alveolar-bone thickness, and height of the labial alveolar bone.

The thickness of the labial cortical bone from 6 mm below the CEJ to the root apex; the thickness of the labial cancellous bone at the root apex; the total thickness of the alveolar bone at the root apex; the area of labial cortical bone; the total area of labial alveolar bone; and the height of the labial alveolar bone were positively correlated with labial inclination of the mandibular central incisors (P < 0.05) (Fig 3).

## Discussion

For patients with skeletal class III malocclusion who undergo compensation or decompensation treatment, the mandibular central incisors often have to be moved to achieve a more satisfactory facial shape and bite. However, excessive tooth movement can lead to iatrogenic sequelae [4]. In clinical practice, it is not feasible to perform CBCT for every patient. Therefore, it is of great clinical significance to be able to predict the morphology of the alveolar bone around the mandibular central incisor from the inclination of that incisor.

In this study, we discovered that, where there were differences in the alveolar-bone height on the labial side of the mandibular central incisors in patients with low-angle, skeletal class III malocclusion, the highest values were in the labial-inclination group, followed by those in the upright and lingual-inclination groups. There were no statistically significant differences in alveolar-bone heights on the lingual side among the three groups. These results coincide with those of the study by Choi et al. [8], in which greater labial inclination of the mandibular incisors were correlated with a smaller alveolar bone height on the labial side of the teeth. This may be because the alveolar bone is thinner on the labial side of the mandibular central incisors than on the lingual side; that is, as the root is close to the alveolar bone on the labial side, the height of the alveolar bone on the labial side is more sensitive to an increased labial inclination of the incisors.

The thickness and area of the cortical bone on the labial side of the mandibular central incisors in patients with low-angle, skeletal class III malocclusion were greater in the labial-inclination group than those in the upright and lingual-inclination groups. These variables were positively correlated with the degree of labial inclination of the mandibular central incisors. However, there were no statistically significant differences in the thickness or areas of the cortical bone on the lingual side among the different groups. Hsu [9] discovered a correlation between alveolar-bone inclination and labial inclination of the tooth; the correlation coefficient was 0.643 on the lingual side and 0.977 on the labial side. This may be why the alveolar bone exhibits different degrees of irregularity on the labial and lingual sides. In addition, there were no differences in the thickness of the alveolar bone on the lingual side of the mandibular central incisors with different degrees of labial inclination, which may be related to a physiological compensation of the human body.

In this study, the total alveolar-bone thickness at the mandibular central incisor was positively correlated with the degree of labial inclination of the tooth, which is similar to the results of Sun et al. [10] in another study of patients with skeletal class III malocclusion. However, Yu Q [11] and others presented opposite findings, in the form of a negative correlation between the degree of labial inclination of the mandibular central incisor and the total alveolar-bone thickness at the root apex. We believe that the reason for the divergence of views is that Yu Q and others did not limit the sagittal facial type of the patients to skeletal class III malocclusion. Mandibular compensation differs between sagittal bone types, which may lead to different trends in total alveolar-bone thickness at the root apex. Therefore, the inclination of mandibular incisors should be studied according to different sagittal bone types for improved analysis of the changes in alveolar-bone thickness.

Based on the results of this study, we provide several suggestions for different clinical situations to guide orthodontists in designing treatment plans to avoid orthodontic risks.

Patients with a lingual inclination of the mandibular incisors in this study had a thinner supporting bone around the root apex than patients without such an inclination, especially in the total alveolar bone and the alveolar bone on the labial side of the root apex. Therefore, we recommend that, for such patients, lingual crown inclination and labial movement of the root should be avoided when the anterior teeth are adducted, to prevent labial root resorption and fenestration when the root is close to the cortical bone.

In the patients with mandibular protrusion and labial inclination of the mandibular incisors, our measurements revealed that the alveolar bone at the root apex on the labial side was thicker than that of patients with a lingual inclination. This provided a large bone-support volume for retraction of the anterior teeth by tilting and movement. During orthodontic treatment, the majority of teeth undergo complex displacement, dominated by the tilting movement; therefore, the mandibular central incisors will move to the center of the basal bone, which is conducive to the long-term stability of their position [12]. Therefore, compared with lingually inclined mandibular central incisors, orthodontic movement of those with labial inclinations are relatively easy and safe to perform.

Compared with previous studies, the measuring equipment and methods used in this study were more accurate, improving the reliability of the results. On the one hand, previously, the morphology of the alveolar bone was mainly evaluated with two-dimensional X-ray imaging. Because of the phase problem of X-ray imaging, it has limited accuracy when used to measure the morphology of the alveolar bone around the anterior teeth [5, 6]. However, CBCT can provide accurate information about the labial and lingual sides of the alveolar bone by revealing the deformation and superposition of the teeth [7].

On the other hand, in the past, the locations of the incisors were usually detected by measuring the thickness of the alveolar bone at the root apex without distinguishing between cortical and cancellous bone [13]. In this study, we improved the measurement methods of Mao and other researchers [14, 15]: we evaluated the alveolar-bone morphology at the mandibular central incisors by measuring the thicknesses and areas of the cancellous and cortical bone at the level of the CEJ; 3, 6, and 9 mm below the CEJ; midway between the CEJ and the root apex; and at the root apex. Compared with past methods, we refined the measurement range and increased the observation angle to provide an all-round picture of the heterogeneous changes in alveolar-bone morphology. In addition, researchers such as Casanova-Sarmiento evenly divided the distance from the CEJ to the root apex and compared the alveolar-bone thickness between different groups at the same levels [16, 17]. The limitation of their relative approach is that the distances differ depending on the size of the teeth, which will affect the measurements.

By using more accurate equipment and improving the measurement methods, our results serve as an accurate guide for clinical practice. Further optimization is needed in terms of the CBCT scanner, fixed-point accuracy, and sample size and type.

First, as the voxel size of CBCT decreases, the measurement accuracy increases [18]. However, a decrease in voxel size will also lead to an increased radiation dose [19]. The voxel size of CBCT used in this study was 0.3 ×0.3 × 0.3 mm, which may have limited the accuracy of evaluation of the alveolar bone. However, Fuhrmann et al. [20] considered that quantitative analysis in CT imaging was feasible when the minimum thickness of the alveolar bone was 0.5 mm. That threshold is larger than the voxel size in this study; hence, from a clinical point of view, the measurement accuracy in this study was satisfactory.

Second, the fixed-point accuracy also directly affects the accuracy of the research results. In this study, we mainly used vision-based measurement, a time-efficient method widely used to study the alveolar bone and upper airways [18, 21]. With this method, the reference points are easily determined by using the interfaces of structures with different densities. However, when the reference point is located at the junction of two structures with similar densities, it is much more difficult to select a suitable area [22]. For such points, the accuracy and repeatability of the gray value-assisted method may be higher [23]. At present, the gray value-assisted method involves the use of two software programs (ImageJ and Excel), and the determination of reference points is a relatively tedious process. To be feasible for clinical practice, this process should be simplified or automated as a software module [24].

Finally, in terms of sample size, we only selected patients with low-angle, skeletal class III malocclusion in this study. Patients with a mean angle and those with a high angle should be included in future for a more comprehensive understanding of the effect of labial inclination of the mandibular central incisors on the morphology of the alveolar bone. At the same time, the small sample included in this study limited the reproducibility of the results. Future research should include more patients to improve the statistical power and more effectively determine the correlation between different variables.

## Conclusion

In patients with low-angle, skeletal class III malocclusion, the morphology of the alveolar bone on the labial but not on the lingual side of the mandibular central incisor was statistically significantly correlated with the labial inclination of those teeth. We suggest that, prior to orthodontic treatment of such patients, clinicians should evaluate the morphological characteristics of the alveolar bone at the mandibular incisors according to its inclination to prevent adverse reactions during treatment.

## Supporting information

S1 File This is the STROBE checklist. (PDF)
