# Supplementary material for: Relationship between alveolar-bone morphology at the mandibular incisors and their inclination in adults with low-angle, skeletal class III malocclusion—A retrospective CBCT study
Source: PLoS One. 2022 Mar 1;17(3):e0264788. doi: 10.1371/journal.pone.0264788 (PMC8887743; doi:10.1371/journal.pone.0264788)
Supplement: S1 File — (PDF) [file pone.0264788.s001.pdf]

STROBE Statement—checklist of items that should be included in reports of observational studies

	Item No.	Recommendation	Page No.	Relevant text from manuscript
**Title and abstract**	1	(*a*) Indicate the study's design with a commonly used term in the title or the abstract	2	A research study
		(*b*) Provide in the abstract an informative and balanced summary of what was done and what was found	2	**Materials and methods:** The CBCT images of 60 patients with low-angle, skeletal class III malocclusion were divided into lingual-inclination, upright, and labial-inclination groups. The height of the alveolar bone and the thickness and area of the cortical, cancellous, and total alveolar bone were measured separately on each side of the mandibular central incisors. **RESULTS**: The thickness of the labial cortical bone from 6 mm below the cementoenamel junction (CEJ) to the root apex; the thickness of the labial cancellous bone at the root apex; the total thickness of the alveolar bone at the root apex; the area of labial cortical bone; the total area of labial alveolar bone; and the height of the labial alveolar bone were highest in the labial-inclination group (all P<0.05). All these variables were positively correlated with the labial inclination of the mandibular central incisors (all P<0.05). There were no statistical differences between the groups for any of the measurements on the lingual side of the teeth (P>0.05).
**Introduction**				
Background/rationale	2	Explain the scientific background and rationale for the investigation being reported	2-3	Researchers overwhelmingly agree that the alveolar bone of the mandibular central incisors in patients with skeletal class III malocclusion is thinner than that in patients with skeletal classes I and II malocclusion. [1.2] Compensatory or decompensatory treatment for skeletal class III malocclusion usually requires extensive movement of the mandibular central incisor. However, the range of orthodontic tooth movement and bone remodeling that can be achieved is restricted by the alveolar-bone morphology; which is known as the "anatomical boundary." [3] Beyond this boundary, the alveolar bone may not undergo complete remodeling, resulting in contact of the root with the cortical bone. Such contact may lead to fenestration, dehiscence, root resorption, and an increased need for orthodontic anchorage. [4] Therefore, the thickness of the alveolar bone of the mandibular central

				incisors should be evaluated prior to orthodontic treatment.<br>Recent research in the field has mainly focused on the influence of different bone types on the thickness of the alveolar bone, or the influence of the change in incisal inclination before and after orthodontic treatment on the morphology of the alveolar bone. However, such studies are mostly limited to the apical area, and most measurements do not distinguish between cortical and cancellous bone. There have been few studies on the effect of labial inclination of the mandibular incisors on alveolar-bone thickness without orthodontic treatment, and there is a lack of data on the effect of cortical- and cancellous-bone area on alveolar-bone thickness. The morphology of the alveolar bone is traditionally evaluated with two-dimensional X-ray imaging, although such evaluation is limited around the anterior teeth. [5.6] In recent years, the application of cone-beam computed tomography (CBCT) has been expanding in the field of dentistry and is regarded as an accurate tool for positioning and measurement. [7] It is a reliable source of data for clinical indicators that are difficult to evaluate, such as alveolar-bone height and thickness.
Objectives	3	State specific objectives, including any prespecified hypotheses	3	We aimed to expand on those results by using CBCT to measure the thickness and height of the alveolar bone around the mandibular central incisors in patients with low-angle, skeletal class III malocclusion with different labial inclinations. The purpose was to determine whether the alveolar-bone thickness and height around the root of the mandibular central incisors differ based on labial inclination.
**Methods**				
Study design	4	Present key elements of study design early in the paper	3	The patients were divided into three groups according to the mandibular central incisor-mandibular plane (L1-MP) angle: the lingual-inclination group, L1-MP < 85.6° (20 cases); the upright group, L1-MP 85.6°–99.6° (20 cases); and the labial-inclination group, L1-MP > 99.6° (20 cases). Patient information is summarized in Table 1, and there were no differences in any of the indicators except for labial inclination.
Setting	5	Describe the setting, locations, and relevant dates, including periods of recruitment, exposure, follow-up, and data collection	3	The researchers retrospectively screened CBCT images that were archived in the First Hospital of Shanxi Medical University from June 2020 to November 2021. The study participants were 34 men and 26 women, age between 18 and 35 years, with an average age of 22 ± 3.93 years.

Participants	6	(*a*) *Cohort study*—Give the eligibility criteria, and the sources and methods of selection of participants. Describe methods of follow-up<br>*Case-control study*—Give the eligibility criteria, and the sources and methods of case ascertainment and control selection. Give the rationale for the choice of cases and controls<br>*Cross-sectional study*—Give the eligibility criteria, and the sources and methods of selection of participants	3	Case-control study—The researchers retrospectively screened CBCT images that were archived in the Stomatology Department of the First Hospital of Shanxi Medical University from June 2020 to June 2021. Sixty-five patients with low-angle, skeletal class III malocclusion were screened, and thirty patients were selected in this study. The study participants were 17 men and 13 women, aged from 18 to 35 years, with an average age of 22.4 ± 4.6 years. The inclusion criteria were as follows: (1) skeletal class III malocclusion, -4°≤ ANB ≤ 1°; (2) MP-FH < 28°; (3) between 15 and 35 years of age; (4) no history of orthodontic treatment; (5) no periodontal disease; (6) no anterior-dentition defects or hyperdontia; (7) no history of anterior-tooth trauma; (8) complete root development, no obvious root resorption, no obvious curvature of the roots of the anterior teeth, and no history of incisal root-canal treatment; (9) crowding of the lower arch < 4 mm; and (10) no facial asymmetry or cleft lip and/or palate.<br>The patients were divided into three groups according to the mandibular central incisor-mandibular plane (L1-MP) angle: the lingual-inclination group, L1-MP < 85.6° (20 cases); the upright group, L1-MP 85.6°–99.6° (20 cases); and the labial-inclination group, L1-MP > 99.6° (20 cases). Patient information is summarized in Table 1, and there were no differences in any of the indicators except for labial inclination.
		(b) Cohort study—For matched studies, give matching criteria and number of exposed and unexposed<br>Case-control study—For matched studies, give matching criteria and the number of controls per case		
Variables	7	Clearly define all outcomes, exposures, predictors, potential confounders, and effect modifiers. Give diagnostic criteria, if applicable	3	(1) skeletal class III malocclusion, -4°≤ ANB ≤ 1°; (2) MP-FH < 28°; (3) between 15 and 35 years of age; (4) no history of orthodontic treatment; (5) no periodontal disease; (6) no anterior-dentition defects or hyperdontia; (7) no history of anterior-tooth trauma; (8) complete root development, no obvious root resorption, no obvious curvature of the roots of the anterior teeth, and no history of incisal root-canal treatment; (9) crowding of the lower arch < 4 mm; and (10) no facial asymmetry or cleft lip and/or palate.
Data sources/	8*	For each variable of interest, give sources of data	4-5	Locating the measurement planes for three-dimensional reconstruction. A:

measurement		and details of methods of assessment (measurement). Describe comparability of assessment methods if there is more than one group		Coronal slice; B: sagittal slice; C: axial slice; D: reconstructed image. The planes are represented as three differently colored lines: red, sagittal plane; blue, axial plane; and green, coronal plane.<br>Step 1: the blue line was adjusted on both the coronal and sagittal slices to overlap with the cementoenamel junction (CEJ) line, to obtain the axial slice.<br>Step 2: in the axial slice, the intersection of the red and green lines was adjusted to the center of the pulp cavity of the cutting plane of the mandibular central incisor, and the image was rotated until the intersection of the red line and the measured tooth was the shortest.<br>Step 3: the sagittal slice was rotated until the green line passed through the root apex and the midpoint of the CEJ. The coronal slice was rotated until the red line passed through the midpoints of the incisal edge and root apex.<br>Finally, the sagittal slice of the desired tooth position was obtained.<br>Measurement items<br>The reference lines and landmark points were determined as follows (Fig 2). First, the long axis of the tooth was defined as the line connecting the midpoint of the CEJ and the root apex. Next, five reference lines perpendicular to the dental long axis were established at the following positions: 3 mm, 6 mm, and 9 mm below the CEJ; the midpoint between the CEJ and root apex; and the root apex. Finally, the landmarks were obtained at the points where each reference line intersected with the medial and lateral sides of the cortical bone.
Bias	9	Describe any efforts to address potential sources of bias	5	All patients were examined by two surveyors on the same computer. Paired t-tests were performed and intraclass correlation coefficients (ICCs) were calculated to assess systematic and random errors. There were no significant differences between the two measurements. The ICC between the two surveyors indicated high reliability (ICC, 0.95). The average of the two measurements was used for each patient.
Study size	10	Explain how the study size was arrived at	3	The size of the population was predetermined by means of power analysis in IBM SPSS Statistics for Windows, version 22.0 (IBM Corp., Armonk, NY, USA).

	With a 1:1:1 group allocation ratio, a total sample size of 60 patients would yield more than 80% power (actual power, 0.944) to detect significant differences with a 0.5138 effect size at the α=0.05 significance level among the three groups.

Continued on next page

Quantitative variables	11	Explain how quantitative variables were handled in the analyses. If applicable, describe which groupings were chosen and why	5	All data were statistically analyzed by using IBM SPSS Statistics. Variables with a normal distribution were expressed as means ± standard deviations. The thickness, height, and area of the alveolar bone in the mandibular central incisor were compared among the three groups of patients by using one-way analysis of variance, and the least significant difference t-test was used for pairwise comparison between groups. The correlation between labial inclination and alveolar-bone morphology of the mandibular central incisor was evaluated by employing the Spearman correlation test. Results were deemed statistically significant when P < 0.05.
Statistical methods	12	(a) Describe all statistical methods, including those used to control for confounding		All data were statistically analyzed by using IBM SPSS Statistics. Variables with a normal distribution were expressed as means ± standard deviations. The thickness, height, and area of the alveolar bone in the mandibular central incisor were compared among the three groups of patients by using one-way analysis of variance, and the least significant difference t-test was used for pairwise comparison between groups. The correlation between labial inclination and alveolar-bone morphology of the mandibular central incisor was evaluated by employing the Spearman correlation test. Results were deemed statistically significant when P < 0.05.
		(b) Describe any methods used to examine subgroups and interactions		
		(c) Explain how missing data were addressed		No data loss issues
		(d) Cohort study—If applicable, explain how loss to follow-up was addressed<br>Case-control study—If applicable, explain how matching of cases and controls was addressed<br>Cross-sectional study—If applicable, describe analytical methods taking account of sampling strategy	3	Case-control study—The patients were divided into three groups according to the mandibular central incisor-mandibular plane (L1-MP) angle: the lingual-inclination group, L1-MP < 85.6° (20 cases); the upright group, L1-MP 85.6°–99.6° (20 cases); and the labial-inclination group, L1-MP > 99.6° (20 cases). Patient information is summarized in Table 1, and there were no differences in any of the indicators except for labial inclination.
		(e) Describe any sensitivity analyses		No exist any sensitivity analyses
Results				
Participants	13*	(a) Report numbers of individuals at each stage of study—eg numbers potentially eligible, examined for eligibility, confirmed eligible, included in the study, completing follow-up, and analysed	3	approximately 20 cases for each group.
		(b) Give reasons for non-participation at each stage		Excluded: No satisfied the inclusion criteria consent

Excluded: No informed consent

(c) Consider use of a flow diagram

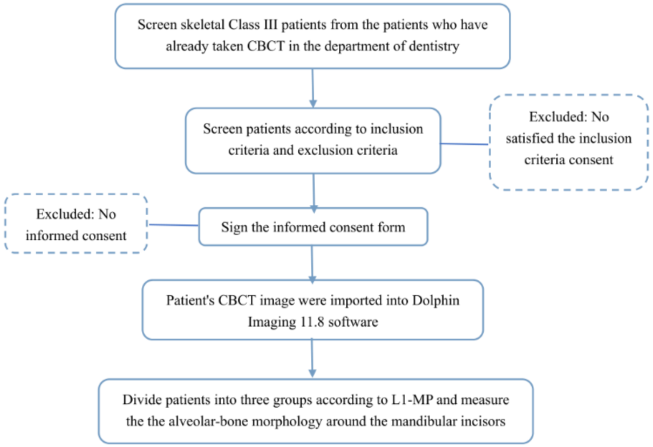

Descriptive data | 14* | (a) Give characteristics of study participants (eg demographic, clinical, social) and information on exposures and potential confounders | 3-4

Table 1. Patient characteristics in different groups

**Characteristics**	**Lingual-inclination group（n=20）**	**Upright group（n=20）**	**Labial-inclination group（n=20）**	**F**	**P**
**Sex(F/M)**	12/8	8/12	6/14	3.801	0.150
**Age(years)**	20.40±4.16	21.95±4.12	22.75±3.37	1.876	0.163
**ANB（°）**	-2.81±1.21	-2.32±2.15	-1.69±0.72	2.868	0.065
**FMA（°）**	19.23±2.00	19.03±2.00	17.94±1.49	2.814	0.068
**L1-MP（°）**	79.47±2.59	89.78±2.33[a]	104.74±3.04[ab]	452.758	＜0.001**

Values presented as mean ± standard deviation.

ANB, subspinale-nasion-supramentale angle; FMA, angle between the Frankfort horizontal plane and the mandibular plane; L1-MP, mandibular central incisor-mandibular plane angle. ** Statistically significant at P < 0.001.

(b) Indicate number of participants with | 0

		missing data for each variable of interest		
		(c) Cohort study—Summarise follow-up time (eg, average and total amount)		
Outcome data	15*	Cohort study—Report numbers of outcome events or summary measures over time		
		Case-control study—Report numbers in each exposure category, or summary measures of exposure		20 cases for each group.
		Cross-sectional study—Report numbers of outcome events or summary measures		
Main results	16	(a) Give unadjusted estimates and, if applicable, confounder-adjusted estimates and their precision (eg, 95% confidence interval). Make clear which confounders were adjusted for and why they were included	5	95% confidence interval
		(b) Report category boundaries when continuous variables were categorized	3	Case-control study—The patients were divided into three groups according to the mandibular central incisor-mandibular plane (L1-MP) angle: the lingual-inclination group, L1-MP < 85.6° (20 cases); the upright group, L1-MP 85.6°–99.6° (20 cases); and the labial-inclination group, L1-MP > 99.6° (20 cases). Patient information is summarized in Table 1, and there were no differences in any of the indicators except for labial inclination.
		(c) If relevant, consider translating estimates of relative risk into absolute risk for a meaningful time period		

Continued on next page

Other analyses	17	Report other analyses done—eg analyses of subgroups and interactions, and sensitivity analyses	5	The thickness, height, and area of the alveolar bone in the mandibular central incisor were compared among the three groups of patients by using one-way analysis of variance, and the least significant difference t-test was used for pairwise comparison between groups. The correlation between labial inclination and alveolar-bone morphology of the mandibular central incisor was evaluated by employing the Spearman correlation test. Results were deemed statistically significant when P < 0.05.
Discussion				
Key results	18	Summarise key results with reference to study objectives	6-8	The thickness of the labial cortical bone from 6 mm below the cementoenamel junction (CEJ) to the root apex; the thickness of the labial cancellous bone at the root apex; the total thickness of the alveolar bone at the root apex; the area of labial cortical bone; the total area of labial alveolar bone; and the height of the labial alveolar bone were highest in the labial-inclination group (all P<0.05). All these variables were positively correlated with the labial inclination of the mandibular central incisors (all P<0.05). There were no statistical differences between the groups for any of the measurements on the lingual side of the teeth (P>0.05).
Limitations	19	Discuss limitations of the study, taking into account sources of potential bias or imprecision. Discuss both direction and magnitude of any potential bias	11	First, although CBCT yields higher-quality measurements than traditional equipment, it has certain limitations. As the voxel size of CBCT decreases, the measurement accuracy increases.[21] However, a decrease in voxel size will also lead to an increased radiation dose.[22] The voxel size of CBCT used in this study was 0.3 ×0.3 × 0.3 mm, which may have limited the accuracy of evaluation of the alveolar bone. However, Fuhrmann et al. [23] considered that quantitative analysis in CT imaging was feasible when the minimum thickness of the alveolar bone was 0.5 mm. That threshold is larger than the voxel size in this study; hence, from a clinical point of view, the measurement accuracy was satisfactory.<br>Second, the fixed-point accuracy also directly affects the accuracy of the research results. In this study, we mainly used vision-based measurement, a time-efficient method widely used to study the alveolar bone and upper airways. [21,24] With this method, the reference points are easily determined by using the interfaces of structures with different densities. However, when the reference point is located at the junction of two structures with similar densities, it is much more difficult to select a suitable area.[25] For such points, the accuracy and repeatability of the gray value-assisted method may be higher.[26] At present, the gray value-assisted method involves the use of two software programs (ImageJ and Excel), and the determination of reference points is a relatively tedious process. To be feasible for clinical practice, this process should be simplified or automated as a software module.[27]<br>Finally, in terms of sample size, we only selected patients with low-angle, skeletal class III

				malocclusion in this study. Patients with a mean angle and those with a high angle should be included in future for a more comprehensive understanding of the effect of labial inclination of the mandibular central incisors on the morphology of the alveolar bone. At the same time, the small sample included in this study limited the reproducibility of the results. Future research should include more patients to improve the statistical power and more effectively determine the correlation between different variables.
Interpretation	20	Give a cautious overall interpretation of results considering objectives, limitations, multiplicity of analyses, results from similar studies, and other relevant evidence	9	In this study, we discovered that, where there were differences in the alveolar-bone height on the labial side of the mandibular central incisors in patients with low-angle, skeletal class III malocclusion, the highest values were in the labial-inclination group, followed by the upright and lingual-inclination groups. There was no statistically significant difference in alveolar-bone height on the lingual side among the three groups. These results coincide with those of the study by Choi et al.,[8] in which greater labial inclination of the mandibular incisors were correlated with a lower alveolar bone on the labial side of the teeth. This may be because the alveolar bone is thinner on the labial side of the mandibular central incisors than on the lingual side; that is, as the root is close to the alveolar bone on the labial side, the height of the alveolar bone on the labial side is more sensitive to an increased labial inclination of the incisors.<br>The thickness and area of the cortical bone on the labial side of the mandibular central incisors in patients with low-angle, skeletal class III malocclusion were greater in the labial-inclination group than in the upright and lingual-inclination groups. These variables were positively correlated with the degree of labial inclination of the mandibular central incisors. However, there were no statistically significant differences in the thickness and area of the cortical bone on the lingual side among the different groups. Hsu[9] discovered a correlation between alveolar-bone inclination and labial inclination of the tooth; the correlation coefficient was 0.643 on the lingual side and 0.977 on the labial side. This may be why the shape of the alveolar bone exhibits differences in regularities on the labial and lingual sides. In addition, there was no difference in the thickness of the alveolar bone on the lingual side of the mandibular central incisors with different degrees of labial inclination, which may be related to a physiological compensation of the human body; that is, regardless of the location of the teeth, the shape of the alveolar bone around the tooth remains relatively constant.<br>In this study, the total alveolar-bone thickness in the mandibular central incisor was positively correlated with the degree of labial inclination of the tooth, which is similar to the

				results of Sun et al. [10] in another study of patients with skeletal class III malocclusion. However, Yu Q[11] and others arrived at the opposite conclusion: that there is a negative correlation between the degree of labial inclination of the mandibular central incisor and total alveolar-bone thickness at the root apex. We believe that the reason for the divergence of views is that Yu Q and others did not limit the sagittal facial type of the patients to skeletal class III malocclusion. Mandibular compensation differs between sagittal bone types, which may lead to different trends in total alveolar-bone thickness at the root apex. Therefore, the inclination of mandibular incisors should be studied according to different sagittal bone types for improved analysis of the changes in alveolar-bone thickness.
Generalisability	21	Discuss the generalisability (external validity) of the study results	11-12	in terms of sample size, we only selected patients with low-angle, skeletal class III malocclusion in this study. Patients with a mean angle and those with a high angle should be included in future for a more comprehensive understanding of the effect of labial inclination of the mandibular central incisors on the morphology of the alveolar bone. At the same time, the small sample included in this study limited the reproducibility of the results. Future research should include more patients to improve the statistical power and more effectively determine the correlation between different variables.
Other information				
Funding	22	Give the source of funding and the role of the funders for the present study and, if applicable, for the original study on which the present article is based		None

*Give information separately for cases and controls in case-control studies and, if applicable, for exposed and unexposed groups in cohort and cross-sectional studies.

Note: An Explanation and Elaboration article discusses each checklist item and gives methodological background and published examples of transparent reporting. The STROBE checklist is best used in conjunction with this article (freely available on the Web sites of PLoS Medicine at http://www.plosmedicine.org/, Annals of Internal Medicine at http://www.annals.org/, and Epidemiology at http://www.epidem.com/). Information on the STROBE Initiative is available at www.strobe-statement.org.
